# An empirical investigation into the impact of winner’s curse on estimates from Mendelian randomization

**DOI:** 10.1093/ije/dyac233

**Published:** 2022-12-27

**Authors:** Tao Jiang, Dipender Gill, Adam S Butterworth, Stephen Burgess

**Affiliations:** BHF Cardiovascular Epidemiology Unit, Department of Public Health and Primary Care, University of Cambridge, Cambridge, UK; Heart and Lung Research Institute, University of Cambridge, Cambridge, UK; Department of Epidemiology and Biostatistics, School of Public Health, Imperial College London, London, UK; Chief Scientific Advisor Office, Research and Early Development, Novo Nordisk, Copenhagen, Denmark; BHF Cardiovascular Epidemiology Unit, Department of Public Health and Primary Care, University of Cambridge, Cambridge, UK; Heart and Lung Research Institute, University of Cambridge, Cambridge, UK; British Heart Foundation Centre of Research Excellence, University of Cambridge, Cambridge, UK; National Institute for Health Research Blood and Transplant Research Unit in Donor Health and Behaviour, University of Cambridge, Cambridge, UK; Health Data Research UK Cambridge, Wellcome Genome Campus and University of Cambridge, Cambridge, UK; BHF Cardiovascular Epidemiology Unit, Department of Public Health and Primary Care, University of Cambridge, Cambridge, UK; Heart and Lung Research Institute, University of Cambridge, Cambridge, UK; MRC Biostatistics Unit, University of Cambridge, Cambridge, UK

**Keywords:** Instrumental variables, winner’s curse, bias, Mendelian randomization, genome-wide association studies, sample overlap

## Abstract

**Introduction:**

Genetic associations for variants identified through genome-wide association studies (GWASs) tend to be overestimated in the original discovery data set as, if the association was underestimated, the variant may not have been detected. This bias, known as winner’s curse, can affect Mendelian randomization estimates, but its severity and potential impact are unclear.

**Methods:**

We performed an empirical investigation to assess the potential bias from winner’s curse in practice. We considered Mendelian randomization estimates for the effect of body mass index (BMI) on coronary artery disease risk. We randomly divided a UK Biobank data set 100 times into three equally sized subsets. The first subset was treated as the ‘discovery GWAS’. We compared genetic associations estimated in the discovery GWAS to those estimated in the other subsets for each of the 100 iterations.

**Results:**

For variants associated with BMI at *P *<* *5 × 10^–8^ in at least one iteration, genetic associations with BMI were up to 5-fold greater in iterations in which the variant was associated with BMI at *P *<* *5 × 10^–8^ compared with its mean association across all iterations. If the minimum *P*-value for association with BMI was *P *=* *10^–13^ or lower, then this inflation was <25%. Mendelian randomization estimates were affected by winner’s curse bias. However, bias did not materially affect results; all analyses indicated a deleterious effect of BMI on coronary artery disease risk.

**Conclusions:**

Winner’s curse can bias Mendelian randomization estimates, although its practical impact may not be substantial. If avoiding sample overlap is infeasible, analysts should consider performing a sensitivity analysis based on variants strongly associated with the exposure.

Key Messages

- Winner’s curse bias occurs in Mendelian randomization investigations when there is overlap between the data set in which the genetic variants were chosen and the data set in which genetic associations were estimated.
- Our empirical investigation indicates that winner’s curse can substantially affect genetic associations, with associations inflated by 1.5–5 times for variants at a *P*-value close to the typical selection threshold of *P* <5×10^–8^.
- However, in our analysis of the effect of body mass index on coronary artery disease risk, overall Mendelian randomization estimates were broadly similar whether there was overlap between the data sets or not.
- Winner’s curse bias can be mitigated by selecting variants based on a more stringent significance threshold but this concern should be balanced against potential low power due to selecting fewer variants.

## Introduction

Mendelian randomization is an epidemiological approach in which genetic variants are used as instrumental variables to assess the existence and potential magnitude of the causal effect of an exposure on an outcome.^1^^,^^2^ Due to the nature of Mendelian inheritance, there is inherent randomness in the transmission of genetic variants from parent to offspring.^3^^,^^4^ This randomness has been shown to hold approximately at a population level for many common genetic variants.^5^^,^^6^ It is therefore possible to treat genetic variants associated with a modifiable trait as unconfounded proxies for the effect of altering that trait, thus mimicking treatment allocation in a randomized–controlled trial.^7^ This makes Mendelian randomization a flexible and credible approach to make causal inferences for a wide range of exposure–outcome pairs. With the rapid expansion of publicly available summary statistics from genome-wide association studies (GWASs) over the recent decade,^8^ Mendelian randomization analyses using such statistics have become increasingly popular.^9^

The concept of winner’s curse originates from auctions in which multiple bidders each have different private estimates on the value of the item for sale.^10^ Under the assumption that all bids are unbiased estimates of the true value of the item but with error, the winner’s bid price will generally be an overestimate of the true value, as the final selling price is the highest bid. This upwards bias in the winner’s bid compared with the true value is known as winner’s curse. In GWASs, the estimated associations of the reported significant variants for a trait are likely to be upwards biased, as they are identified based on a statistical significance threshold—only the ‘winning’ variants are reported as significant.^11^ Whereas winner’s curse bias will be most severe for associations with the trait under investigation, genetic associations with any variable correlated with the trait will also tend to be overestimated. In particular, in the context of Mendelian randomization, genetic associations with an outcome will likely also be overestimated in the discovery GWAS data set for an exposure, as exposures and outcomes are typically associated due to confounding.

This bias can have a direct impact on Mendelian randomization estimates calculated using association estimates derived from the discovery GWAS. With a single genetic variant, the Mendelian randomization estimate can be expressed as the ratio of the genetic association with the outcome divided by the genetic association with the exposure.^12^ With multiple genetic variants, the standard combined estimate (the inverse-variance weighted estimate) is a weighted mean of these ratio estimates calculated for each variant.^13^ Hence, winner’s curse in the ‘exposure’ association estimates would be expected to result in a ‘deflation’ in the Mendelian randomization estimate, whereas winner’s curse in the ‘outcome’ association estimates would be expected to result in an ‘inflation’ in the Mendelian randomization estimate.

Winner’s curse can be alleviated by selecting genetic variants and estimating genetic associations in non-overlapping data sets. However, it may not be possible to find distinct sets of summary statistics from independent data sets for the same trait. The degree of bias from winner’s curse depends on various factors and is typically worse for genetic variants with associations close to the statistical significance threshold. Several methods have been proposed to correct for winner’s curse bias.^14–19^ However, these methods can be overly conservative and result in a loss in power or assume an underlying distribution that may not hold.

A related phenomenon in Mendelian randomization is weak instrument bias, which arises due to chance associations of the genetic variants with confounders.^20^^,^^21^ Even if a genetic variant is a valid instrumental variable (i.e. has no true association with confounders), correlations of the variant with confounders will not be exactly zero. If a genetic variant is strongly associated with the exposure, then bias due to chance associations with the confounders is negligible. However, if a genetic variant is weakly associated with the exposure, then bias due to chance imbalances in the distribution of a confounder can be non-negligible. For a ‘one-sample’ Mendelian randomization analysis, in which the same data set is used for estimating the genetic associations with the exposure and the outcome, these chance correlations affect associations with both the exposure and the outcome in a related way, and bias (known as weak instrument bias) is ‘towards the observational association’ between the exposure and outcome. For a ‘two-sample’ Mendelian randomization analysis, in which genetic associations with the exposure and outcome are obtained from independent samples, these chance correlations will differ between the data sets and so affect associations with the exposure and the outcome independently, and weak instrument bias is ‘towards the null’.^22^ The magnitude of weak instrument bias depends on the instrument strength, which can be estimated as the F-statistic from regression of the exposure on the genetic variants.^23^^,^^24^

Both winner’s curse and weak instrument bias are finite-sample biases, in that they reduce towards zero when the sample size gets large. However, whereas the expected magnitude of bias due to weak instruments can be approximated (and is typically slight when the genetic variants are associated with the exposure at a conventional genome-wide level of statistical significance, *P *<* *5 × 10^–8^), the expected magnitude of bias due to winner’s curse in a Mendelian randomization investigation is typically unclear. This is important to study, as any analysis must balance concerns about winner’s curse and weak instrument bias against other biases, such as bias from instrument invalidity due to pleiotropy.

Here, we provide an empirical investigation into the magnitude of bias from winner’s curse and weak instruments in an applied Mendelian randomization analysis considering the effect of body mass index (BMI) on coronary artery disease (CAD) risk. We picked this example because there is previous evidence supporting a causal relationship^25^^,^^26^ and several hundred genetic variants have been found to be associated with BMI in previous GWASs, meaning that we can compare genetic association estimates for a large number of variants. We took data from UK Biobank, which we randomly split into three equally sized subsets to consider different scenarios in which the genetic variants were chosen based on their associations in one subset (the ‘discovery GWAS’) and associations with the exposure and outcome were estimated in either the same subset or a different subset. We repeated this splitting procedure to investigate the distribution of Mendelian randomization estimates. To compare the magnitude of bias in different situations, we also considered Mendelian randomization estimates obtained only using genetic variants that were statistically strongly associated with the exposure, as well as estimates obtained only using genetic variants that were not fully consistent in their association with the exposure across data sets. We conclude by discussing the relevance of these findings for bias in other Mendelian randomization investigations and the implications for how Mendelian randomization investigations should be performed.

## Methods

### Study population and outcomes

All analyses were performed in the UK Biobank data set—a prospective cohort study of ∼500 000 UK residents aged 40–69 years. We took data on 367 644 unrelated participants of European ancestries, as ascertained by a mixture of self-report and genetic information following quality-control procedures previously described.^27^ BMI was calculated as weight in kilogrammes divided by height in metres squared. CAD was defined using International Classification of Diseases, Tenth Revision (ICD-10) codes as ICD-10 code I21–I25 or self-reported data from touchscreen questionnaire or interview with a nurse practitioner (reporting a ‘heart attack’ or ‘myocardial infarction’ in Field 6150 or 20002, or reporting a coronary angioplasty, coronary artery bypass graft or triple heart bypass in Field 20004).

### Discovery GWAS and obtaining Mendelian randomization estimates

We split UK Biobank participants into three equally sized groups at random, which we refer to as Group A (the discovery GWAS group), Group B and Group C. Using data from Group A, we performed a genome-wide association analysis for BMI adjusting for age, sex and the first 10 genomic principal components. Variants were filtered for a minor allele frequency of >0.01% and an INFO score (an imputation quality metric) of >0.4.^28^ Variants passing these filters and reaching the conventional genome-wide significance threshold of *P *<* *5 × 10^–8^ were selected as instruments. In order to ensure that selected variants are mutually independent, we clumped the selected variants into loci using a distance threshold of 1 megabase and a correlation threshold of *r*^2^<0.01 estimated within Group A, to ensure no two selected variants are too close or too highly correlated with each other. We selected the variant with the smallest *P*-value in each locus to create our discovery set.

For each variant in our discovery set, we estimated its genetic association with BMI in each of Groups A, B and C by using linear regression adjusting for age, sex and the first 10 genomic principal components. We also calculated genetic associations with CAD risk in each group by using logistic regression with the same covariate adjustment. We note that the associations with BMI in Group A are the same as those obtained in the discovery GWAS. We combined these summarized genetic association estimates across variants to obtain Mendelian randomization estimates using the random-effects inverse-variance weighted method.^29^ This estimate represents the log odds ratio for CAD per 1-kg/m^2^ higher genetically predicted BMI. This entire process was repeated for 100 iterations taking different random splits of the original data set in each iteration.

### Analysis set-up

We considered five scenarios, corresponding to different situations in which winner’s curse and weak instrument bias should or should not occur (Table 1). We use the term ‘overlap’ to indicate that genetic associations are estimated in the same participants as the discovery GWAS (Group A) and one-sample or two-sample to indicate whether genetic associations with the exposure and outcome are estimated in the same or different groups, respectively.

**Table 1:** Summary of scenarios considered in the empirical analysis

Scenario	Group genetic associations estimated in for …		Winner’s curse affects genetic association estimates for …		One-sample?
	Exposure	Outcome	Exposure	Outcome	
1	B	C			
2	A	B	✓		
3	B	A		✓	
4	B	B			✓
5	A	A	✓	✓	✓

In each scenario, Group A was used as the discovery genome-wide association study (GWAS).

1. No overlap, two-sample: genetic associations with BMI are estimated in Group B and genetic associations with CAD in Group C, i.e. we have three distinct data sets for discovery, exposure associations and outcome associations. This represents a scenario in which winner’s curse will not occur and weak instrument bias will be towards the null. We treat this as the reference scenario for comparison purposes. This scenario is sometimes called ‘three-sample Mendelian randomization’.
2. Overlap with exposure, two-sample: genetic associations with BMI are estimated in Group A and genetic associations with CAD in Group B, i.e. we have overlapping discovery and exposure data sets. Winner’s curse will affect genetic associations with BMI, but not with CAD risk, so Mendelian randomization estimates should be deflated. Weak instrument bias will be towards the null.
3. Overlap with outcome, two-sample: genetic associations with BMI are estimated in Group B and genetic associations with CAD in Group A, i.e. we have overlapping discovery and outcome data sets. Winner’s curse will affect genetic associations with CAD risk, but not with BMI, so Mendelian randomization estimates should be inflated. Weak instrument bias will be towards the null.
4. No overlap, one-sample: genetic associations with BMI and CAD were both estimated in Group B, i.e. we have no overlap between the discovery and estimation data sets, but genetic associations with the exposure and outcome were obtained in the same data set. Winner’s curse will not occur and weak instrument bias will be towards the observational association between BMI and CAD risk (which is positive).
5. Overlap with exposure and outcome, one-sample: genetic associations with BMI and CAD were both estimated in Group A, i.e. all analyses were performed on the same data set. This represents a scenario in which winner’s curse will occur for genetic associations with both BMI and CAD risk, so Mendelian randomization estimates will be subject to both inflation and deflation; it is not clear what the net effect will be. Weak instrument bias will be towards the observational association between BMI and CAD risk.

In addition to assessing winner’s curse in the Mendelian randomization estimates, we also considered the amount of winner’s curse in the genetic associations for individual variants, defined as the percentage difference in the mean beta-coefficient across iterations for which the association was significantly associated with BMI (at *P *<* *5 × 10^–8^) divided by the mean beta-coefficient across all 100 iterations:


$$\%\;\text{difference}=\frac{\left(\text{Mean beta when significant for BMI}-\text{Mean beta across all iterations}\right)}{\text{Mean beta across all iterations}}\times 100.$$

We define the absolute difference similarly:


Absolute difference=Mean beta when significant for BMI-Mean beta across all iterations.

Note that these measures are defined for genetic associations with BMI and with CAD risk, but in both cases statistical significance is judged based on the genetic associations with BMI. This reflects that in a Mendelian randomization investigation, variants are selected based on their associations with the exposure, not the outcome.

In secondary analyses, we considered two further strategies for selecting variants. First, we considered a stricter statistical significance threshold for variant selection of *P *<* *5 × 10^–11^. Only variants meeting this threshold were included in the Mendelian randomization analyses. There was no specific reason for the choice of 5 × 10^–11^; we simply wanted to illustrate the impact of winner’s curse at a different selection threshold. Second, we noted which genetic variants were selected into the discovery set for all of the 100 random splits of the original data set. Loci from which a variant was selected in all 100 random splits were removed from these analyses. The motivation of this ‘no full replication’ strategy is to assess the impact of winner’s curse for a trait when only variants with relatively weaker evidence of association are available.

We also compared results from the inverse-variance method to those from two methods that correct for winner’s curse bias. We investigated the three scenarios with overlap (and hence are affected by winner’s curse bias): Scenarios 2 (‘overlap with outcome, two-sample’), 3 (‘overlap with outcome, two-sample’) and 5 (‘overlap with exposure and outcome, one-sample’) for the primary choice of variants. We considered the false-discovery-rate inverse-quantile-transformation (FIQT) method^14^ and the debiased inverse-variance weighted (DIVW) method.^19^ The FIQT method explicitly corrects for the genetic association test statistics (Z-scores) having non-zero mean by implementing a multiple testing correction. The DIVW estimate is obtained by multiplying the inverse-variance weighted estimate by a debiasing factor that accounts for bias due to weak instruments and variant selection (which the original authors call ‘screening’). The inverse-variance weighted estimate can be calculated as: where ${\;\hat{\beta }}_{Xj}$ is the beta-coefficient for the association of the *j*-th genetic variant with the exposure, ${\hat{\beta }}_{Yj}$ is the beta-coefficient for the association of the *j*-th genetic variant with the outcome, $se({\hat{\beta }}_{Yj})$ is the standard error of this beta-coefficient and summation is across the genetic variants. The DIVW estimate is calculated as: where $se({\;\hat{\beta }}_{Xj})$ is the standard error of the association of the *j*-th genetic variant with the exposure.


$$\frac{\sum_{j}{\;\hat{\beta }}_{Yj}{\;\hat{\beta }}_{Xj}{se({\hat{\beta }}_{Yj})}^{-2}}{\sum_{j}{{\;\hat{\beta }}_{Xj}}^{2}{se({\hat{\beta }}_{Yj})}^{-2}}$$

$$\frac{\sum_{j}{\;\hat{\beta }}_{Yj}{\;\hat{\beta }}_{Xj}{se({\hat{\beta }}_{Yj})}^{-2}}{\sum_{j}\left({{\hat{\beta }}_{Xj}}^{2}-{se({\hat{\beta }}_{Xj})}^{2}\right){se({\hat{\beta }}_{Yj})}^{-2}}$$

To implement the FIQT method, we input Z-scores for genetic associations with the exposure in Group A into the FIQT function and used the FIQT-corrected Z-scores to scale the beta-coefficients for the outcome (Scenario 2), for the exposure (Scenario 3) or for both the exposure and outcome (Scenario 5). As an example, if the original Z-score is +6 and the FIQT-corrected Z-score is +5.7, then we multiplied the relevant beta-coefficients for that variant by a factor of 5.7/6 = 0.95 (so a 5% reduction). We still selected variants using the original *P*-values, not the FIQT-corrected *P*-values. This was done to maintain comparability of estimates and to ensure that any reduction in bias was due to the FIQT method and not simply because fewer variants were selected. However, in practice, the FIQT method could be used to facilitate a more stringent selection of variants.

Genetic association analyses were performed using SNPTEST v2^30^ and Mendelian randomization analyses were performed using the MendelianRandomization package^31^ in R v3.6.1.^32^

## Results

The mean age of the 367 644 participants at baseline was 57.2 years and 54.1% of participants were female. The mean BMI was 27.4 kg/m^2^ and there were 29 330 CAD events. Overall, variants in 359 loci were associated with BMI at a genome-wide significance threshold (*P *<* *5 × 10^–8^) in Group A for at least one iteration of the random splitting procedure. Of these, 7 loci contained a variant significantly associated with BMI in all 100 iterations, whereas variants in the remaining 352 loci were significantly associated with BMI in some iterations but not others. The median number of variants selected in each iteration was 39.

**Figure 1:**
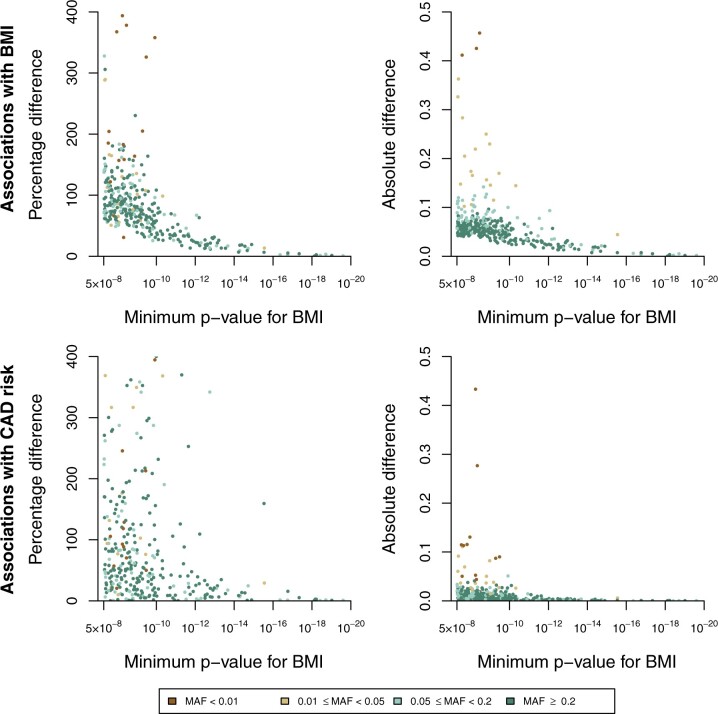
Scatter plot showing variants selected as associated with body mass index (BMI) in at least one iteration. Estimates represent percentage and absolute differences in the mean beta-coefficient estimate in Group A between its mean value across all iterations and its mean across only those iterations for which it was associated with BMI at *P* <5 × 10^–8^ calculated for associations with BMI (top row) and coronary artery disease (CAD) risk (bottom row), plotted against its minimum *P*-value for BMI across iterations. Only one variant per locus is plotted. Nine variants had a minimum *P*-value of <10^–20^; percentage and absolute differences were close to zero for these variants. Percentage differences for associations with CAD risk exceeded 400 for 42 variants (maximum value was 4766); these points are not shown on these axes (see Supplementary Figure S1, available as Supplementary data at *IJE* online). Colours correspond to minor allele frequencies

Inflation of genetic associations due to winner’s curse for individual variants is illustrated in Figure 1. For genetic variants that had a minimum *P*-value for association with the exposure across iterations of ∼5 × 10^–8^, the percentage difference for the association with BMI in iterations when it was statistically significant (*P *<* *5 × 10^–8^) in its association with the exposure compared with its mean value across all iterations varied from 50% to 400%, suggesting that beta-coefficients for associations with the exposure were on average between 1.5 and 5 times too large in these data sets. For genetic variants that had a minimum *P*-value of ∼1 × 10^–13^, the percentage difference varied from 10% to 25% (i.e. 1.1- to 1.25-fold inflation due to winner’s curse). A similar pattern was observed in the absolute difference in associations, which reduced in magnitude considerably as the minimum *P*-value decreased. Inflation was also observed in the genetic associations with CAD risk. On the percentage difference scale, several variants had inflated associations with CAD risk up to and beyond 400% (Supplementary Figure S1, available as Supplementary data at *IJE* online). However, some of these percentage differences were large because the mean association with CAD risk across all iterations was close to zero. On the absolute difference scale, associations with CAD risk were less inflated than those with BMI.

When assessing the relationship between winner’s curse bias and minor allele frequency (Figure 1, colour of points), there is a clear association between absolute bias and minor allele frequency (Spearman’s correlation coefficient for BMI associations = –0.550, *P *<* *0.001; for CAD risk = –0.299, *P *<* *0.001), with large values of bias being associated with rarer variants. There are several reasons for this, including the greater magnitude of genetic associations with rarer variants and the greater variability of genetic associations with rarer variants. There was a weaker association between relative bias and minor allele frequency (Spearman’s correlation coefficient for BMI associations = –0.231, *P *<* *0.001; for CAD risk = –0.088, *P *=* *0.096), suggesting that rarer variants are also more likely to have larger biases on the relative scale.

Mendelian randomization estimates are summarized in Table 2. In the primary analyses using all variants, all Mendelian randomization estimates were positive and had *P*<0.05 in each iteration. The median estimate from Scenario 1 (no overlap, two-sample) was 0.0829, corresponding to an odds ratio of 1.086 per 1-kg/m^2^ increase in genetically predicted BMI, which is similar to what has been observed previously.^26^ As expected, the median estimate from Scenario 2 (overlap with exposure, two-sample) deflated to 0.0720 (corresponding to an odds ratio of 1.075) and the median estimate from Scenario 3 (overlap with outcome, two-sample) inflated to 0.0950 (corresponding to an odds ratio of 1.100). Judging by the differences in median estimates, winner’s curse in genetic associations with the outcome affected Mendelian randomization estimates similarly strongly to winner’s curse in genetic associations with the exposure.

**Table 2:** Summary of primary analysis results

Scenario		Median estimate	Mean estimate	Mean standard error	Standard deviation of estimates
1	No overlap, two-sample	0.0829	0.0829	0.0201	0.0143
2	Overlap with exposure, two-sample	0.0720	0.0724	0.0164	0.0132
3	Overlap with outcome, two-sample	0.0950	0.0948	0.0204	0.0152
4	No overlap, one-sample	0.0913	0.0893	0.0204	0.0157
5	Overlap with exposure and outcome, one-sample	0.0863	0.0847	0.0158	0.0123

Estimates represent log odds ratios for coronary artery disease per 1-kg/m^2^ increase in genetically predicted body mass index. For each scenario, we report the median and mean estimates across 100 iterations, the mean standard error of estimates and the standard deviation of estimates. All estimates are obtained from the random-effects inverse-variance weighted method.

Comparing Scenario 1 (no overlap, two-sample) and Scenario 4 (no overlap, one-sample) shows the impact of weak instrument bias separate from winner’s curse. The median estimate from Scenario 5 was 0.0913 (corresponding to an odds ratio of 1.096), suggesting that the impact of weak instrument bias is less than the impact of winner’s curse in this example. Scenario 5 (overlap with exposure and outcome, one-sample) is the most complex scenario to predict, as here we have winner’s curse in the genetic associations with both exposure and outcome, plus weak instrument bias in the direction of the observational association. Winner’s curse with the exposure would be expected to deflate estimates, whereas winner’s curse with the outcome and weak instrument bias would be expected to inflate estimates. The median estimate from Scenario 5 was 0.0863 (corresponding to an odds ratio of 1.090), indicating that the competing biases approximately cancelled out.

**Figure 2:**
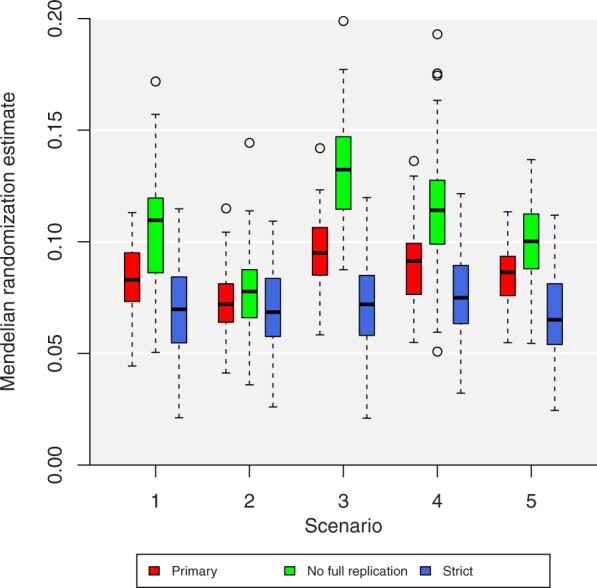
Box plots of primary and secondary results. Estimates represent log odds ratios for coronary artery disease per 1-kg/m^2^ increase in genetically predicted body mass index. Box plot indicates the lower quartile, median and upper quartile of estimates; error bars represent the range of estimates up to 1.5 times the interquartile range. Outliers outside this range are plotted separately. All estimates are obtained from the random-effects inverse-variance weighted method. Primary = all variants associated with BMI at *P*<5×10^–8^. No full replication = same as primary, but excluding all loci containing a variant associated with BMI at *P* <5×10^–8^ in all 100 iterations. Strict = all variants associated with BMI at *P* <5×10^–11^

Results from the secondary analyses considering different strategies for selecting variants are presented in Figure 2. First, we see that median estimates in Scenario 1 (no overlap, two-sample) differ slightly between the three strategies. There is no systematic reason why these differences occur, other than that they are based on different variants that may affect BMI in different ways. In this example, estimates using the strict strategy (blue boxes) are generally slightly smaller in magnitude than those from the primary strategy using all variants (red boxes), whereas estimates using the ‘no full replication’ strategy (green boxes) are slightly larger.

Compared with Scenario 1, the pattern of median estimates in Scenarios 2–4 is broadly the same for each variant selection strategy. Median estimates are deflated in Scenario 2 and inflated in Scenarios 3 and 4. The degree of variation due to bias was around two to three times larger for the ‘no full replication’ strategy and much lower for the strict strategy. In the primary and ‘no full replication’ strategies, inflation was greater in Scenario 3 (due to winner’s curse) than in Scenario 4 (due to weak instrument bias). In contrast, in the strict strategy, inflation was slightly greater in Scenario 4 than Scenario 3, although the difference was minimal. Whereas there was some difference of median estimates in Scenario 5, this was not substantial for any of the strategies.

Results from the FIQT and DIVW methods, which correct for winner’s curse bias, are shown in Table 3. Results were broadly similar from all three methods. Mean and median estimates from the FIQT method were slightly greater than those from the inverse-variance weighted method in Scenario 2 and slightly less in Scenario 3; in both cases, this represents a reduction in winner’s curse bias, but the reduction was not substantial. Mean and median estimates were nearly equal in Scenario 5. This may be because in Scenario 5, associations with both the exposure and outcome are subject to bias, and correcting for bias in both sets of associations has an overall neutral effect on estimates. Mean and median estimates from the DIVW method were slightly greater than those from the inverse-variance weighted method in all scenarios. We conclude that if variant selection is based on a genome-wide significance threshold (*P *<* *5 × 10^–8^), winner’s curse correction methods may have more utility in helping select variants that are robustly associated with the exposure, rather than correcting estimates based on a given set of variants.

**Table 3:** Summary of results for winner’s curse correction methods

Scenario	Method	Median estimate	Mean estimate	Mean standard error	Standard deviation of estimates
2	Inverse-variance weighted	0.0720	0.0724	0.0164	0.0132
2	FIQT	0.0733	0.0737	0.0167	0.0134
2	DIVW	0.0735	0.0739	0.0166	0.0135
3	Inverse-variance weighted	0.0950	0.0948	0.0204	0.0152
3	FIQT	0.0933	0.0929	0.0201	0.0149
3	DIVW	0.0985	0.0980	0.0210	0.0157
5	Inverse-variance weighted	0.0863	0.0847	0.0158	0.0123
5	FIQT	0.0864	0.0847	0.0159	0.0124
5	DIVW	0.0881	0.0864	0.0159	0.0126

Results from Scenarios 2 (overlap with exposure, two-sample), 3 (overlap with outcome, two-sample) and 5 (overlap with exposure and outcome, one-sample) for inverse-variance weighted, false-discovery-rate inverse-quantile-transformation (FIQT) and debiased inverse-variance weighted (DIVW) methods. Estimates represent log odds ratios for coronary artery disease per 1-kg/m^2^ increase in genetically predicted body mass index. For each method, we report the median and mean estimates across 100 iterations, the mean standard error of estimates and the standard deviation of estimates.

## Discussion

In this paper, we have explored the impact of winner’s curse bias on Mendelian randomization estimates for the effect of BMI on CAD risk. By dividing the UK Biobank study into three groups at random a large number of times, we were able to compare the distribution of Mendelian randomization estimates in scenarios in which the discovery and estimation data sets were distinct and scenarios in which they overlapped. When the discovery data set overlapped with the data set used for estimating genetic associations with the exposure, Mendelian randomization estimates were typically deflated, whereas when the discovery data set overlapped with the data set for estimating genetic associations with the outcome, Mendelian randomization estimates were typically inflated.

We were able to compare the magnitude of bias from winner’s curse to bias from weak instrument bias. In the primary analysis including all genome-wide significant variants (*P *<* *5 × 10^–8^), bias from winner’s curse was greater in magnitude. Bias from both weak instruments and winner’s curse was substantially lower when only including variants associated with BMI at a stricter significance threshold (*P *<* *5 × 10^–11^) and bias was substantially greater when excluding variants consistently strongly associated with BMI. In the latter case, winner’s curse bias was again greater in magnitude than weak instrument bias. In the former case, both biases were slight, although weak instrument bias was slightly larger than winner’s curse bias. Additionally, we showed that winner’s curse for individual variant associations can be very substantial in practice, with inflation in beta-coefficients for the exposure of 50–400% for variants that only just achieved the GWAS significance threshold (*P *≈* *5 × 10^–8^). However, the degree of bias dropped off sharply for variants associated with the exposure at a stricter significance threshold.

Despite bias from winner’s curse and weak instruments, Mendelian randomization evidence for a positive effect of BMI on CAD risk was obtained in all primary analysis iterations. Although it would be unwise to conclude that winner’s curse bias is never substantial in Mendelian randomization analyses based on a single empirical analysis, some important lessons can be learned from this work. First, the magnitude of bias from winner’s curse depends sharply on the statistical strength of genetic associations. If genetic variants are strongly associated with the exposure (in terms of statistical strength of evidence), then there is little uncertainty as to whether they will be selected or not from the discovery GWAS and so winner’s curse bias is minimal. In contrast, if genetic associations with the exposure are close to the GWAS significance threshold then bias will be more substantial. Second, bias from winner’s curse was generally greater in magnitude than weak instrument bias but the two biases had a roughly similar order of magnitude in our empirical example.

Although winner’s curse bias in genetic associations with the exposure is unwelcome, theoretically in isolation it will not lead to inflated Type 1 error rates for Mendelian randomization estimates. Although the magnitude of a Mendelian randomization estimate depends on associations with the exposure and outcome, its significance only depends on the genetic associations with the outcome. This is because the null hypothesis that the Mendelian randomization estimate is zero is achieved exactly when the genetic association with the outcome is zero; if the genetic association with the outcome is zero, then the Mendelian randomization estimate will be zero regardless of the genetic association with the exposure. Additionally, winner’s curse in outcome association estimates typically inflates Mendelian randomization estimates, which is more worrying as it can lead to false-positive findings, whereas deflation in estimates is typically conservative. Hence, winner’s curse bias is a more serious problem in practice when it affects genetic associations with the outcome. Fortunately, as shown in our example, the absolute bias in genetic association estimates due to winner’s curse is lower for associations with the outcome compared with associations with the exposure, as genetic variants are selected based on their associations with the exposure. However, the proportional bias can still be large.

For simplicity, we have only considered scenarios in which there is either complete or no overlap between the discovery and estimation data sets. If there is partial overlap between data sets, winner’s curse bias will be less substantial. Given their international collaborative nature, many GWAS consortia have some degree of overlap. Additionally, the same participants may have been recruited into multiple studies. Hence, even if genetic associations appear to come from non-overlapping data sets, participant overlap may be non-zero in practice.

The ideal situation is that Mendelian randomization analyses operate using a ‘three-sample’ design, in which discovery GWASs, exposure associations and outcome associations are assessed in non-overlapping data sets.^33^ However, in practice, it is rarely feasible to find three distinct large samples of participants that are sufficiently similar to combine into a single analysis (e.g. same ancestry group). Even if it is possible, the loss in sample size (and hence power) from not including the discovery GWAS in the estimation of genetic associations may be unwelcome. Additionally, differences in participant characteristics between samples may mean that genetic variants discovered in one data set are not the most relevant predictors of the exposure in a second data set. Another possibility for avoiding winner’s curse bias is cross-validation, whereby a large data set is divided into discovery and estimation subsets. This can be performed efficiently by dividing the data set into tenths and performing discovery in 90% of the data and then obtaining association estimates in the remaining tenth.^34^ By repeating this procedure for each tenth of the data separately and combining results, participant overlap can be avoided while minimizing loss of power. A further suggestion is to perform a sensitivity analysis which only includes variants that are statistically strongly associated with the exposure (say, those that achieve *P *<* *10^–11^). However, this may again lead to an unwelcome loss of power. Alternatively, a method that attempts to correct for winner’s curse bias can be employed,^14–19^ although our investigations suggested these approaches may be more effective when used to aid the selection of variants (as indicated by the reduction in bias when using a stricter selection threshold) rather than to reduce bias for a given choice of variants.

Avoiding participant overlap between the discovery GWAS and the estimation data set for associations with the outcome is an important factor in determining how to perform a Mendelian randomization analysis.^29^ However, pragmatic choices often have to be made to balance the possibility of a somewhat biased analysis vs the possibility of an uninformative analysis due to low sample size. Additionally, there are several other biases that could affect a Mendelian randomization analysis, the most important of which is pleiotropy (or more generally, violation of instrument validity). Whereas analysts and reviewers should pay attention to all potential sources of bias, pleiotropy is the most critical consideration when assessing the validity of a Mendelian randomization investigation.^29^ Our investigation suggests that winner’s curse is a relevant consideration when deciding how to choose data sets for analysis, but winner’s curse may not bias estimates substantially or affect overall conclusions.

In conclusion, bias due to winner’s curse affects Mendelian randomization estimates and the magnitude of bias in our empirical investigation was similar but generally larger than that from weak instruments. Analysts should carefully consider the possibility of avoiding sample overlap between the discovery GWAS and the estimation data set for associations with the outcome, particularly if most genetic variants are close to the statistical significance threshold, as well as performing sensitivity analyses that reduce winner’s curse bias.

## Ethics approval

This research was conducted according to the principles expressed in the Declaration of Helsinki. The UK Biobank cohort has been approved by the North West Multicentre Research Ethics Committee, UK (Ref: 16/NW/0274). Written informed consent has been obtained from all study participants. The current study was approved by the UK Biobank Access Management Board under application 7439. Participants who had withdrawn consent by the time of the analysis were excluded from the data set.

## Supplementary Material

dyac233_Supplementary_Data

## Data Availability

UK Biobank data are available by application at https://www.ukbiobank.ac.uk/enable-your-research/apply-for-access to any *bona fide* researcher.

## References

[1] Lawlor DA , Harbord RM, Sterne JA, Timpson N, Davey Smith G. Mendelian randomization: using genes as instruments for making causal inferences in epidemiology. Stat Med 2008;27:1133–63. 17886233 10.1002/sim.3034

[2] Burgess S , Thompson SG. Mendelian Randomization: Methods for Using Genetic Variants in Causal Estimation. Boca Raton, Florida, USA: Chapman & Hall/CRC Press, 2015.

[3] Hingorani A , Humphries S. Nature's randomised trials. Lancet 2005;366:1906–08. 16325682 10.1016/S0140-6736(05)67767-7

[4] Thanassoulis G , O'Donnell CJ. Mendelian randomization: nature's randomized trial in the post-genome era. JAMA 2009;301:2386–88. 19509388 10.1001/jama.2009.812 PMC3457799

[5] Davey Smith G , Lawlor D, Harbord RM, Timpson N, Day I, Ebrahim S. Clustered environments and randomized genes: a fundamental distinction between conventional and genetic epidemiology. PLoS Med 2007;4:e352. 18076282 10.1371/journal.pmed.0040352 PMC2121108

[6] Taylor M , Tansey KE, Lawlor DA, et al Testing the principles of Mendelian randomization: Opportunities and complications on a genomewide scale. *bioRxiv*; doi:10.1101/124362, 7 April 2017, preprint: not peer reviewed.

[7] Swanson SA , Tiemeier H, Ikram MA, Hernán MA. Nature as a trialist? Deconstructing the analogy between Mendelian randomization and randomized trials. Epidemiology 2017;28:653–59. 28590373 10.1097/EDE.0000000000000699 PMC5552969

[8] Visscher PM , Wray NR, Zhang Q et al 10 years of GWAS discovery: biology, function, and translation. Am J Hum Genet 2017;101:5–22. 28686856 10.1016/j.ajhg.2017.06.005 PMC5501872

[9] Burgess S , Scott RA, Timpson NJ, Davey Smith G, Thompson SG; EPIC-InterAct Consortium. Using published data in Mendelian randomization: a blueprint for efficient identification of causal risk factors. Eur J Epidemiol 2015;30:543–52. 25773750 10.1007/s10654-015-0011-z PMC4516908

[10] Bazerman MH , Samuelson WF. I won the auction but don't want the prize. J Conflict Resolut 1983;27:618–34.

[11] Lohmueller KE , Pearce CL, Pike M, Lander ES, Hirschhorn JN. Meta-analysis of genetic association studies supports a contribution of common variants to susceptibility to common disease. Nat Genet 2003;33:177–82. 12524541 10.1038/ng1071

[12] Didelez V , Sheehan N. Mendelian randomization as an instrumental variable approach to causal inference. Stat Methods Med Res 2007;16:309–30. 17715159 10.1177/0962280206077743

[13] Burgess S , Butterworth A, Thompson SG. Mendelian randomization analysis with multiple genetic variants using summarized data. Genet Epidemiol 2013;37:658–65. 24114802 10.1002/gepi.21758 PMC4377079

[14] Bigdeli TB , Lee D, Webb BT et al A simple yet accurate correction for winner's curse can predict signals discovered in much larger genome scans. Bioinformatics 2016;32:2598–603. 27187203 10.1093/bioinformatics/btw303 PMC5013908

[15] Xiao R , Boehnke M. Quantifying and correcting for the winner's curse in genetic association studies. Genet Epidemiol 2009;33:453–62. 19140131 10.1002/gepi.20398 PMC2706290

[16] Zhong H , Prentice RL. Correcting ‘winner's curse’ in odds ratios from genomewide association findings for major complex human diseases. Genet Epidemiol 2010;34:78–91. 19639606 10.1002/gepi.20437 PMC2796696

[17] Mounier N , Kutalik Z, Bias correction for inverse variance weighting Mendelian randomization. *bioRxiv*. doi:10.1101/2021.03.26.437168, 18 December 2021, preprint: not peer reviewed. 37036286

[18] Bowden J , Dudbridge F. Unbiased estimation of odds ratios: combining genomewide association scans with replication studies. Genet Epidemiol 2009;33:406–18. 19140132 10.1002/gepi.20394 PMC2726957

[19] Ye T , Shao J, Kang H. Debiased inverse-variance weighted estimator in two-sample summary-data Mendelian randomization. Ann Stat 2021;49:2079–100. 10.1002/sim.10245 39453381

[20] Nelson CR , Startz R. The distribution of the instrumental variables estimator and its t-ratio when the instrument is a poor one. J Bus 1990;63:S125–40.

[21] Burgess S , Davies NM, Thompson SG. Bias due to participant overlap in two-sample Mendelian randomization. Genet Epidemiol 2016;40:597–608. 27625185 10.1002/gepi.21998 PMC5082560

[22] Pierce BL , Burgess S. Efficient design for Mendelian randomization studies: subsample and 2-sample instrumental variable estimators. Am J Epidemiol 2013;178:1177–84. 23863760 10.1093/aje/kwt084 PMC3783091

[23] Stock JH , Wright JH, Yogo M. A survey of weak instruments and weak identification in generalized method of moments. J Bus Econ Stat 2002;20:518–29.

[24] Burgess S , Thompson SG. Bias in causal estimates from Mendelian randomization studies with weak instruments. Stat Med 2011;30:1312–23. 21432888 10.1002/sim.4197

[25] Hägg S , Fall T, Ploner A et al; European Network for Genetic and Genomic Epidemiology Consortium. Adiposity as a cause of cardiovascular disease: a Mendelian randomization study. Int J Epidemiol 2015;44:578–86. 26016847 10.1093/ije/dyv094 PMC4553708

[26] Larsson SC , Bäck M, Rees JM, Mason AM, Burgess S. Body mass index and body composition in relation to 14 cardiovascular conditions in UK Biobank: a Mendelian randomization study. Eur Heart J 2020;41:221–26. 31195408 10.1093/eurheartj/ehz388 PMC6945523

[27] Astle WJ , Elding H, Jiang T et al The allelic landscape of human blood cell trait variation and links to common complex disease. Cell 2016;167:1415–29.e19. 27863252 10.1016/j.cell.2016.10.042 PMC5300907

[28] Howie BN , Donnelly P, Marchini J. A flexible and accurate genotype imputation method for the next generation of genome-wide association studies. PLoS Genet 2009;5:e1000529. 19543373 10.1371/journal.pgen.1000529 PMC2689936

[29] Burgess S , Davey Smith G, Davies NM et al Guidelines for performing Mendelian randomization investigations. Wellcome Open Res 2019;4:186. 32760811 10.12688/wellcomeopenres.15555.1 PMC7384151

[30] Marchini J , Howie B, Myers S, McVean G, Donnelly P. A new multipoint method for genome-wide association studies by imputation of genotypes. Nat Genet 2007;39:906–13. 17572673 10.1038/ng2088

[31] Yavorska OO , Burgess S. MendelianRandomization: an R package for performing Mendelian randomization analyses using summarized data. Int J Epidemiol 2017;46:1734–39. 28398548 10.1093/ije/dyx034 PMC5510723

[32] R Core Team. R: A Language and Environment for Statistical Computing. Vienna: R Foundation for Statistical Computing, 2021.

[33] Zhao Q , Chen Y, Wang J, Small DS. Powerful three-sample genome-wide design and robust statistical inference in summary-data Mendelian randomization. Int J Epidemiol 2019;48:1478–92. 31298269 10.1093/ije/dyz142

[34] Denault WR , Bohlin J, Page CM, Burgess S, Jugessur A. Cross-fitted instrument: a blueprint for one-sample Mendelian Randomization. PLoS Comput Biol 2022;18:e1010268. 36037248 10.1371/journal.pcbi.1010268 PMC9462731

