# Supplementary material for: An empirical investigation into the impact of winner’s curse on estimates from Mendelian randomization
Source: Int J Epidemiol. 2022 Dec 27;52(4):1209–19. doi: 10.1093/ije/dyac233 (PMC10396423; doi:10.1093/ije/dyac233)
Supplement: dyac233_Supplementary_Data [file dyac233_supplementary_data.pdf]

SUPPLEMENTARY MATERIALS

Supplementary Figure S1: Scatter plot showing all variants selected as associated with body mass index

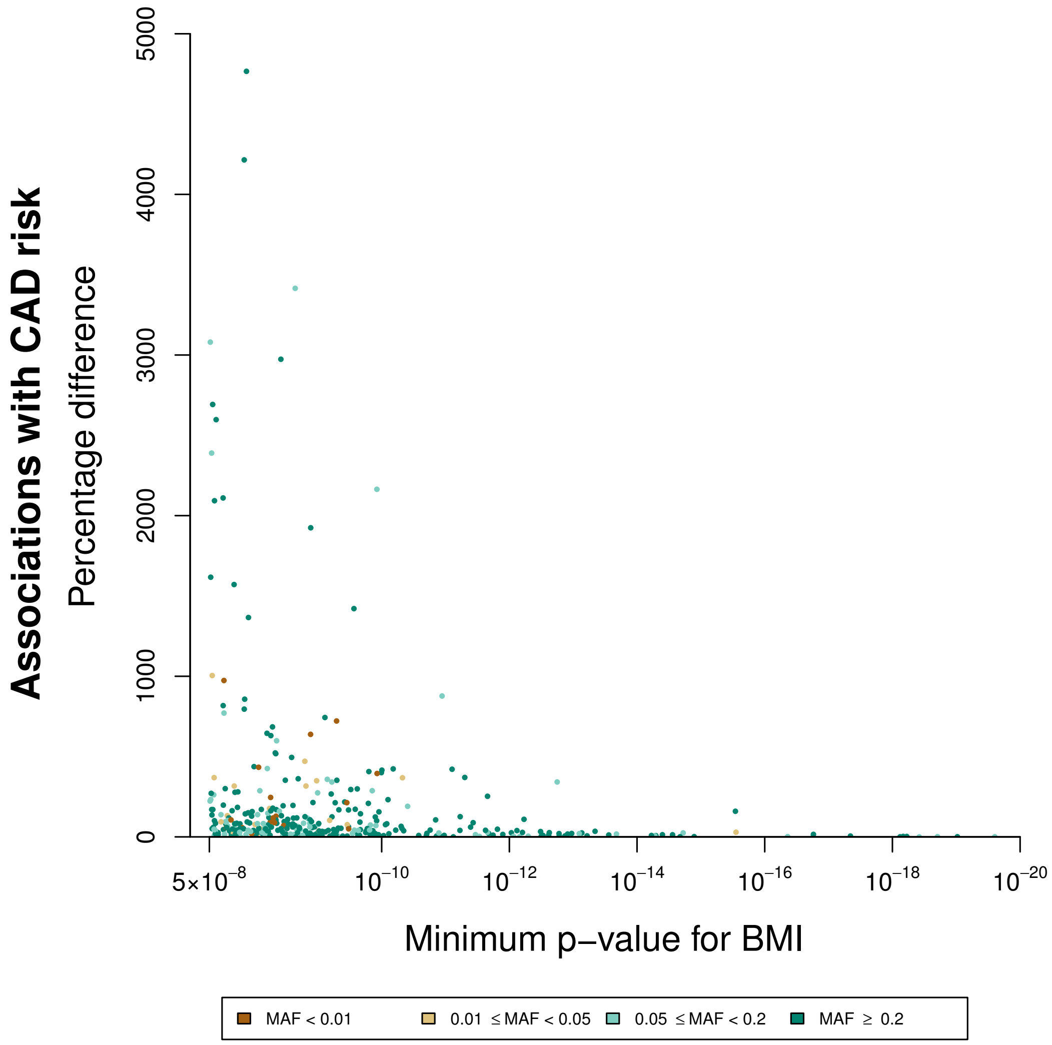

Scatter plot showing variants selected as associated with body mass index (BMI) in at least one iteration: the percentage difference in the beta-coefficient estimate for the association with coronary artery disease (CAD) risk in Group A between its average value across all iterations and its average across only those iterations for which it was significant for BMI, plotted against its minimum p-value for BMI across iterations. Only one variant per locus is plotted. This plot is identical to the bottom-left panel of **Figure 1**, except the y-axis is extended here to accommodate plotting of all points. Colours correspond to minor allele frequencies (MAF).
